# High-Order SNP Combinations Associated with Complex Diseases: Efficient Discovery, Statistical Power and Functional Interactions

**DOI:** 10.1371/journal.pone.0033531

**Published:** 2012-04-19

**Authors:** Gang Fang, Majda Haznadar, Wen Wang, Haoyu Yu, Michael Steinbach, Timothy R. Church, William S. Oetting, Brian Van Ness, Vipin Kumar

**Affiliations:** 1 Department of Computer Science, University of Minnesota, Minneapolis, Minnesota, United States of America; 2 Department of Genetics, Cell Biology, and Development, University of Minnesota, Minneapolis, Minnesota, United States of America; 3 Minnesota Supercomputing Institute, University of Minnesota, Minneapolis, Minnesota, United States of America; 4 Department of Environmental Health Sciences, University of Minnesota, Minneapolis, Minnesota, United States of America; 5 Department of Experimental and Clinical Pharmacology, University of Minnesota, Minneapolis, Minnesota, United States of America; Pennsylvania State University, United States of America

## Abstract

There has been increased interest in discovering combinations of single-nucleotide polymorphisms (SNPs) that are strongly associated with a phenotype even if each SNP has little individual effect. Efficient approaches have been proposed for searching two-locus combinations from genome-wide datasets. However, for high-order combinations, existing methods either adopt a brute-force search which only handles a small number of SNPs (up to few hundreds), or use heuristic search that may miss informative combinations. In addition, existing approaches lack statistical power because of the use of statistics with high degrees-of-freedom and the huge number of hypotheses tested during combinatorial search. Due to these challenges, functional interactions in high-order combinations have not been systematically explored. We leverage discriminative-pattern-mining algorithms from the data-mining community to search for high-order combinations in case-control datasets. The substantially improved efficiency and scalability demonstrated on synthetic and real datasets with several thousands of SNPs allows the study of several important mathematical and statistical properties of SNP combinations with order as high as eleven. We further explore functional interactions in high-order combinations and reveal a general connection between the increase in discriminative power of a combination over its subsets and the functional coherence among the genes comprising the combination, supported by multiple datasets. Finally, we study several significant high-order combinations discovered from a lung-cancer dataset and a kidney-transplant-rejection dataset in detail to provide novel insights on the complex diseases. Interestingly, many of these associations involve combinations of common variations that occur in small fractions of population. Thus, our approach is an alternative methodology for exploring the genetics of rare diseases for which the current focus is on individually rare variations.

## Introduction

Genotype-phenotype association studies, from both targeted and genome-wide data, have contributed to our ability to identify genetic variants that are associated with disease. Although an increasing number of studies have found single-nucleotide polymorphisms (SNPs) that have statistically significant association with diseases, most of them either have small effects on disease risk [1]–[3] or often explain only a small part of the population [4]–[7]. Thus, there has been increased interest in discovering combinations of SNPs that are strongly associated with a phenotype even if each SNP has little or even no individual effect [8]–[12]. Our goal is to discover and study such combinations of SNPs to complement existing approaches for univariate analysis or pathway/network enrichment-based approaches that are built upon univariate statistics [13]–[16]. In particular, as pursued by [17]–[23], we focus on discovering SNP combinations, especially high-order ones beyond size 2, that are strongly associated with a phenotype and yield information on interpretable statistical and functional interactions.

There are two challenges in finding SNP combinations that are highly associated with a phenotype from a large number of SNPs. The first arises from the combinatorial nature of the problem, i.e. there are exponentially increasing number of combinations as the order goes higher. This is even more problematic if a large number of permutation tests are used to correct for multiple hypothesis tests [13], [24], [25]. Given a GWAS dataset with hundreds of thousands of SNPs, even the examination of pair-wise combinations of SNPs is computationally challenging [23], and requires efficient enumeration algorithms [23], [26]–[28] or specialized hardwares [29], [30]. Finding higher order SNP combinations [17], [31] is far more computationally expensive and is out of reach for GWAS datasets. Hence, existing methods mostly explore higher order SNP combinations with datasets that only have tens or few hundreds of SNPs. These methods adopt either brute-force or heuristic-based greedy search. Brute-force approaches such as multifactor dimensionality reduction (MDR [17]), or the combinatorial partitioning method (CPM [19]) can guarantee the completeness of the search, which is important in detecting SNP combinations with weak marginal effects [22]. However, these brute-force approaches can handle only a relatively small number of SNPs (tens or hundreds) [17], [18], [32]. The scalability of recent approaches [33] has been improved to allow searching for size 3 combinations from about 600 SNPs within two hours. However, it is still not capable of efficiently handling focus studies that have thousands of SNPs [34], [35], especially for higher order combinations. Greedy search strategies [36]–[44], although more computationally efficient than brute-force approaches, risk missing significant SNP combinations [11], [12], [23], and rarely discover high-order combinations beyond size 3 [17], and only from datasets containing tens or hundreds of SNPs covering a even smaller number of genes.

The second challenge is that existing approaches for high-order SNP combination searches lack statistical power. Specifically, due to the use of statistics with high degree of freedom [31], [45] and the huge number of hypothesis tested with often limited sample sizes, many high-order combinations of SNPs can be strongly associated with a disease phenotype by random chance, resulting in a high false discovery rate [25]. Some existing approaches [10], [38], [46] use biological pathways or molecular interaction networks as constraints to reduce the number of hypotheses to test and make the interpretation easier. Essentially, a set of SNPs are considered for an association test only if the SNPs are located around the genes that are on a common pathway or interact with each other. A common limitation of such constraint-based approaches is that, they may miss novel SNP combinations that are not on known pathways or interaction subnetworks due to the incompleteness of biological knowledge. Thus, it calls for a quantitative evaluation on trade off between the reduction of search space and the risk of missing informative SNP combinations, and also calls for alternative constraints that are not limited by existing biological knowledge.

In this paper, we aim to address both the above challenges.

To improve computational efficiency, we leverage the discriminative pattern mining framework (DPM, originally proposed [47], [48] in the data mining community for mining market basket data) to efficiently search for high-order SNP combinations from SNP datasets in focused studies with thousands of SNPs. The computational efficiency and scalability of DPM is enhanced by the systematic pruning of the combinatorial search space with anti-monotonic objective functions. A unique advantage of anti-monotonicity-based search over brute-force search is that it can avoid exploring the whole search space (all combinations of SNP genotypes) by pruning a large number of candidates that cannot lead to a sufficiently strong association with a phenotype [48], [49]. We demonstrate that DPM has substantially improved efficiency and scalability on a synthetic and three real datasets with several thousands of SNPs. We observe that most high-order combinations are trivial extensions of their subsets which are not interesting but consume most of the total computation time, however, there are indeed high-order combinations that have discriminative power significantly beyond singleton SNP or low-order SNP combinations.

To improve the statistical power, we study the effect of two strategies that reduce the number of high-order combinations being tested. The first, which does not depend on the use of prior biological knowledge, is to require an increase in discriminative power for a combination over its subsets. We demonstrate that this constraint can reduce the number of hypothesis tests dramatically and thus enable the discovery of significant combinations that would have been missed otherwise. The second strategy, which depends on the known biological knowledge, is to use gene-set (e.g. pathway) constraints within the DPM framework. While this approach has been used in existing work to improve computational efficiency, we quantitatively evaluate its effect on enhancing statistical power in conjunction with the DPM framework.

The improved computational efficiency and statistical power further enables the discovery of significant high-order SNP combinations from the three real datasets and then allows the exploration of functional interactions in high-order SNP combinations. Specifically, we study the functional interactions among the genes covered in high-order SNP combinations with an integrated human functional gene network. We find a positive connection between the increase of discriminative power of a SNP combination over its subsets and the functional coherence among the genes covered in the combination. Such an observation is beyond the disease-specific functional interactions studied by existing work that are based on datasets covering a small number of genes [17] and is supported by the multiple real datasets used in the paper. In addition to this disease-independent biological insight, we also interpret several high-order combinations discovered from the lung cancer [MIM: 211980] dataset and the dataset for studying rejection after kidney transplant, which provide novel insights beyond univariate or low-order SNP-combination analysis. More generally, we find that many significant associations are combinations of common variations that occur in small fractions of population. This suggests an alternative direction for the exploration of the genetics of rare diseases, where the current focus is mainly on analyzing individually rare variations.

## Results

### Three Real Case-control SNP Datasets and a Synthetic Dataset

We use three SNP datasets designed for studying different types of disease phenotypes: (i) short (less than one year) vs. long (greater than three years) survival of multiple myeloma [MIM: 254500] patients [34] (denoted as *Survival*), (ii) acute rejection [MIM: N/A] (within in six months) vs. non-rejection (within eight years) after kidney transplant [22] (denoted as *Kidney*), (iii) lung cancer [MIM: 211980] vs. non-lung cancer (both heavy smokers) [35] (denoted as *Lungcancer*). The three datasets were all collected with a chip [34] targeting 3444 SNPs in 983 genes, representing cellular functions and pathways that may influence disease severity at diagnosis, toxicity, progression or other treatment outcomes. Previous analyses on these three datasets did not reveal statistically significant single SNPs after correcting for multiple hypothesis testing, and this study aims to explore if there are significant (after correcting for multiple hypothesis testing) associations between combinations of SNPs and disease phenotypes, especially high-order combinations (with size greater than 2) that have stronger association beyond single SNPs or low-order combinations.

Preprocessing and quality control steps are described in the method section. Table S1 summarizes the number of SNPs after quality control and the numbers of cases and controls for each of the datasets. More information on these datasets can be found in the original papers. All the datasets are available from the Eastern Cooperative Oncology Group (ECOG) through requests to the operations office (http://www.ecog.org/, accessed 2012 Feb 20). In addition to the three real datasets, We also use a synthetic dataset with 70 cases and 70 controls, 2172 SNPs without differentiation between the cases and controls, and four synthetic high-order SNP combinations of size 3, 4, 5 and 6 respectively, that are associated with case-control groupings. (See the methods section for simulation details).

Note that, the above four datasets have much larger number of SNPs (ranging from 2172 to 3428) than the datasets used in previous studies on high-order SNP interactions (tens or hundreds of SNPs). With these four datasets, we will show that the proposed framework is substantially more efficient and scalable than existing approaches. Although the proposed approach could not directly handle datasets with more than 10,000 SNPs due to the intrinsic computational complexity of high-order SNP combination search, it is worth noting that tag SNP selection [50] techniques can be used to first obtain a set of less redundant SNPs before the use of the proposed approach. In this way, genome-wide studies with hundreds of thousands of SNPs could also be analyzed.

### The Binary Encoding of a SNP and a Combination of SNPs

We use a binary coding scheme of SNP genotypes, where we create three binary columns for each SNP (Figure S1). For a single SNP (*X*) with three genotypes (homozygous minor (mm), heterozygous (Mm) and homozygous major (MM).), we create three binary variables as 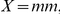 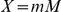 and 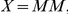 each of which is represented as a binary variable indicating if a person’s genotype for SNP X is *mm*, *mM* or *MM* respectively. Figure S1 illustrates the transformation from categorical encoding to binary encoding. Note that, this is a lossless transformation because it can be mapped back to the original SNP genotypes without ambiguity. As will be shown later, the use of this binary coding is to enable the efficient traversal of the combinatorial search space in the discriminative pattern mining (DPM) framework used in the paper. Although the number of columns increases to three times of the original number of SNPs, we show the DPM framework has substantially better efficiency and scalability than existing approaches that directly search from the categorical SNP variables. It is worth noting that, binary encoding was also leveraged in [33], where the authors commented that, while binary coding may have somewhat weaker power, it does allows the use of efficient enumeration algorithms and the discovery of biologically interesting SNP combinations.

Based on the binary coding for each SNP genotype, a combination of SNPs is essentially a combination of SNP genotypes. For example, for three SNPs *X*, *Y* and *Z*, a combination might be 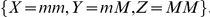 Such a combination is also called a *pattern* in this paper, where we use the terms “pattern”, “combination” and “SNP combination” interchangeably. Following the traditional setup in discriminative pattern analysis, a pattern is said to be *present* in a subject only if the subject’s genotypes match all the SNP genotypes in the pattern, and *absent* otherwise. Thus, a combination of SNP genotypes (multiple SNPs, each contributing one of its genotype) is also encoded as a binary variable (*present* or *absent*). Again, we use this setup to allow DPM to efficiently perform the search of combinatorial pattern space. The frequency of a pattern (the percentage of subjects in which a pattern is present, also called *support*) has a mathematical property named anti-monotonicity, which can be leveraged by DPM to prune most of the combinatorial search space and only investigate those patterns that are more likely to have strong association with a disease phenotype [47], [48] (see methods section).

With this binary encoding of a SNP combination, a 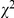 test of the association between any combination and a binary phenotype has a fixed degree of freedom of 1 [33] and is independent of the size of the combination. Here, the goal is to test the association between the present and absent of the SNP combination, under the binary encoding, and a binary phenotype. Note that, other statistical measures can also be used for similar purpose. This also implies that the proposed framework can handle datasets with imbalanced number of cases and controls. The degree of freedom being 1 is an important advantange for high-order SNP combination analysis because most real datasets have a limited number of samples that are insufficient for estimating the association between a combination of larger size and a disease phenotype if the statistical measure in use has a degree of freedom increasing with the size of a combination. The fixed degree of freedom also allow the direct comparison of the statistics (e.g. 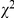 statistic or others) of SNP combinations of different sizes, which is important for quantifying the gain of discriminative power of a SNP combination with respect to its subsets. For example, the size-3 combination 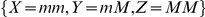 has three size-2 subsets: 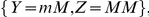 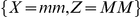 and 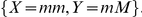


### Illustrative Examples of High-order Discriminative SNP Combinations

**Figure 1:**
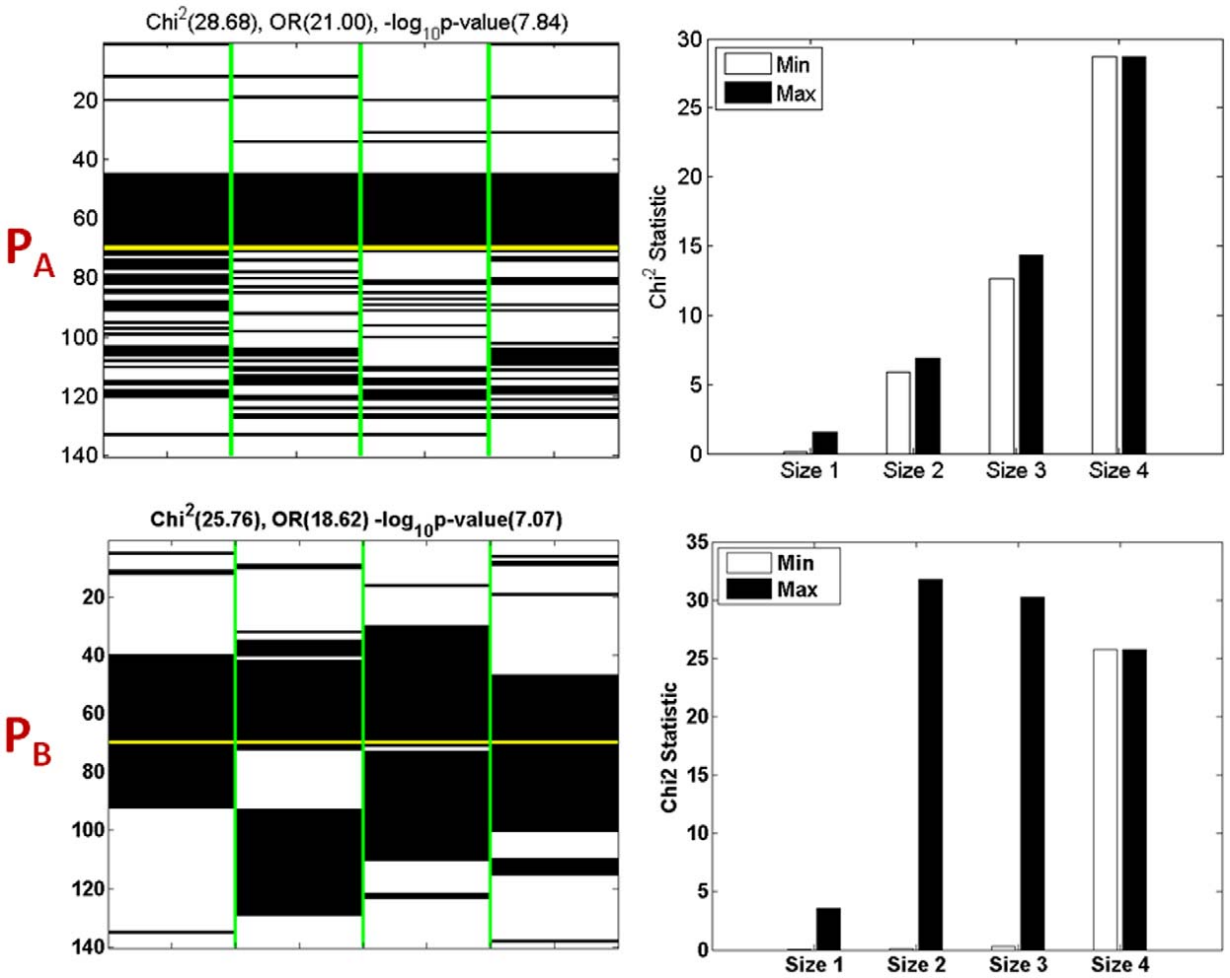
Visualization of the two synthetic SNP-genotype combinations and their high-order association with the two classes. The two subfigures in the left column are the visualization of the genotypes of 4 SNPs separated by vertical green lines, over the 70 cases and 70 controls separated by a horizontal yellow line. The black color indicates present and the white indicates absent, in the binary format described in the method section. The 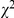 statistic, odds ratios and the 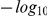 fisher exact test p value of the two combinations are (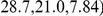 and 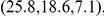 respectively. Each subfigure in the right column contains 4 pairs of bars. For each pair, the unfilled bar and the filled bar indicate the minimal and the maximal 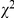 statistics for the size-*i* (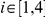) subsets of the combination. The right most pair, both bars are equal since they both denote the 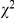 statistic of the SNP combination itself. Another four examples of high-order discriminative SNP combinations of size-36 are shown in Figure S2 with similar description as this figure.

After describing the above binary encoding of a SNP combination, we first illustrate two examples of high-order SNP combination shown in Figures 1 (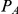 and 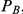 generated with the method developed in [51]) before presenting the efficient search algorithm. 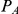 is a pattern containing four SNPs (separated by vertical green lines) over 70 cases and 70 controls, which are separated by a horizontal yellow line (cases top, controls bottom). The black color indicates presence (1′s) and the white indicates absence (0′s) of one of the three genotypes of a SNP. The 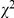 statistic, odds and the 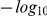 fisher exact test p-value of the synthetic combination (as a binary encoded single variable as described above) are 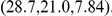 The subfigure in the right column contains 4 pairs of bars. For each pair, the left bar (unfilled) and the right bar (filled) indicate the minimal and the maximal 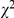 statistics for the size-*i* (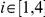) subsets of the combination. For the right most pair, both bars are equal since they both denote the 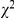 statistic of the SNP combination itself. As shown, the 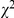 statistic of 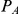 is higher than all of its subsets, which makes 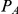 interesting because it provides predictive power beyond that of its subsets. Thus, it is important to discover this high-order pattern as a highly confident predictive rule with an odds ratio of 21, rather than discover its subsets.

Similar to 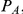 pattern 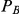 in Figure 1 also has high discriminative power in terms of 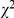 statistic, odds ratios and the 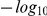. However, in contrast to 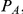 pattern 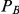 is actually less discriminative than one of its size-2 subset (the first two SNP columns), as reflected by the drop in the 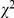 statistic in the right subfigure. Later in this section, we will differentiate these two types of SNP combinations and show that SNP combinations like 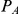 provide more information for the functional interactions among the genes in a SNP combination, while the high discriminative power of patterns like 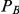 are trivial consequences of their highly differentiating subsets. Figure S2 shows four high-order SNP combinations of size-3 to size-6 (generated with [51]) that we embedded in the synthetic dataset described earlier, all having higher discriminative power than their subsets. Indeed, such interesting high-order SNP combinations also exist in real datasets for studying complex diseases such as cancer, as will be shown in the result section.

### Discovering High-order SNP Combinations that have Strong Association with a Phenotype

With the two discriminative SNP combinations shown in Figure 1 and the additional examples in Figure S2, we now describe how to leverage the discriminative pattern mining (DPM) framework to efficiently search for high-order SNP combinations that have strong association with a disease phenotype. The DPM mining framework was originally proposed in the data mining community to efficiently enumerate combinations of variables and identify those that are highly predictive [52], [53]. DPM builds upon a general search strategy called Apriori [47], which leverages the anti-monotonicity of a special type of objective functions for efficient enumeration of high-order variable combinations (see methods for details). Conceptually, with an objective function that is anti-monotonic, a SNP combination satisfies a threshold on the objective function only if all its subsets satisfies the threshold. In another word, if a combination does not pass a threshold on the objective function, all of its supersets can be pruned in the search space and it is guaranteed that no larger combination that satisfies the threshold would be missed. This is the key difference between Apriori-based combinatorial search and brute-force combinatorial search.

In this study, we leverage a recently developed anti-monotonic objective function 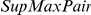 [48] and use it in the Apriori framework to efficiently search for SNP combinations that are discriminative between cases and controls. 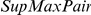 captures the association between a SNP combination and a binary disease phenotype (see the methods section), i.e. the higher 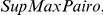 the stronger the SNP combination is associated with the phenotype. The Apriori framework using 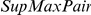 as the objective function is called SMP [48] and has the advantage of handling dense and high dimensional data, which addresses the key challenge in discovering high-order combinations from SNP datasets, i.e. a fixed high density of 33% as a result of the binary encoding of each SNP (Each SNP is represented with three binary columns and the genotype of a sample for each SNP is represented by a 1 in one of the three columns (assuming there is no missing value). Thus, one third of the matrix values are 1′s (a density of 33%).) and a large number of SNPs (high dimensionality). This advantage owes to the effective use of phenotype information in the searching process [48] and is the essence of SMP’s better efficeincy and scalablity over other DPM algorithms.

It is worth noting that Ma et al. [33] is the first that leverages an Apriori-based algorithm [54] (FPC) for the efficient enumeration of SNP combinations. However, FPC does not make use of phenotype information to optimize the search process and thus is much less efficient and less scalable than SMP, as has been shown in [48] on differential gene expression analysis and will also be demonstrated on SNP datasets in the result section of this study. SMP is part of the framework we implement for this study and is available on the paper website (http://vk.cs.umn.edu/HSC/, accessed 2012 Feb 20).

### The DPM Framework has Substantially Better Efficiency and Scalability

We compare the DPM framework with two representative existing tools for high-order SNP combination discovery: MDR [17] (http://www.epistasis.org/software.html) and the framework presented in [33] (denoted as FPC in this paper). For MDR, we used the Java version (http://sourceforge.net/projects/mdr/) and used the standard coding, in which each SNP is represented by a categorical value with three possible values (genotypes). For DPM and FPC, we use the binary coding. FPC requires an input for the parameter 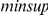 (the minimum frequency of a pattern in the set of cases and controls combined). For comparison purpose, we set a five-hour maximal runtime allowance (Though arbitrary, some threshold needs to be selected for comparison purpose) for all the three techniques. Experiments presented here were run on a Linux machine with 10 Intel(R) Xeon(R) CPUs (2.00GHz) and 100GB memory.

In the synthetic dataset (described in the method section), there are 2172 SNPs. The three frameworks need to search through size-2, size-3, size-4, size-5 and size-6 combinations in order to discover the four embedded patterns of size-3 to 6. After five hours, MDR was still enumerating size-3 SNP combinations, and thus failed to identify the embedded size-4, size-5 and size-6 patterns. FPC could reach size-6 within five hours, but only with a 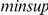 threshold of 0.9 (With a 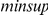 threshold of 0.8, FPC could not finish even in 24 hours.), which is so high that none of the four synthetic patterns were discovered (the frequency of the four embedded patterns are all below 0.25.). In contrast, the run time of SMP on the synthetic dataset is around 4 minutes with a 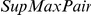 threshold of 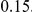 The threshold of 0.15 was chosen such that all the four embedded synthetic SNP combinations can be discovered. At lower threshold, additional discriminative SNP combinations can be discovered (if they exist), but it will take more computational time. In practice, one should use a threshold as low as possible while the computational time is still acceptable (usually decided after some tests). In addition, given a fixed 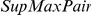 threshold and a fixed number of SNPs, the patterns discovered from a dataset with larger sample size are expected to be more statistically significant in term of false discovery rate. Therefore, given a certain statistical significance cutoff, a lower 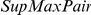 threshold should be used for datasets with larger sample sizes while the computational time is still acceptable.

The discovered SNP combinations are of size 2 to 10, including all the four embedded patterns. The substantially better efficiency of SMP is also observed on the three real datasets, which have 2755–3428 SNPs (Table S1). The substantially better efficiency and scalability of SMP over FPC and MDR is due to the effective use of phenotype information in SMP for pruning combination candidates that are less likely to form a larger discriminative pattern as discussed in the method section (refer to [48] for further details). Indeed, the efficiency of the proposed framework (search as high as size-10 combinations from thousands of SNPs within one hour) is superior to not just MDR and FPC, but also to several other existing approaches which can discover up to size-3 SNP combinations from datasets with hundreds of SNPs [18], [32], [33], [40], [55]. Furthermore, we designed an experiment to test the scalability of SMP with respect to the sample size. We vary the sample size (cases and controls combined) from 140 to 5600 in seven steps (140, 280, 420, 560, 1400, 2800 and 5600) as shown in Figure (see method section for the details of data simulation). The first four steps representing one, two, three and four times of the samples in the first synthetic dataset (used in the comparison with MDR and FPC), respectively. The last three steps correspond to a much larger samples sizes in several thousands that represent the number of samples in most GWAS studies. The running time shown on the y-axis of Figure S3 shows that the computational time of SMP increases approximately in a linear manner with respect to the sample size (recall that the x-axis is not linearly spaced). This agrees with the theoretical time complexity of Apriori-based searching algorithms [47] and indicates that SMP is able to handle datasets with much larger number of samples than the three real datasets used in this paper.

Note that, the synthetic datasets used above (to demonstrate the better efficiency and scalability of DPM over MDR and FPC) are representatives of the three real datasets used in the paper. For datasets with smaller number of SNPs (e.g. tens or hundreds of SNPs), MDR and FPC (as well as other similar approaches) have been compared with other approaches [21], [33] and demonstrated to be scalable (mostly up to size 3 combinations). In this study, we have the specific focus on datasets with thousands of SNPs such as the three real datasets or datasets of tag SNPs selected from genome-wide studies, and we are particularly interested in high-order interaction (its mathematical and statistical properties as well as functional insights). Therefore, we will only use DPM in the rest of the analyses.

### Identifying High-order SNP Combinations with Stronger Association than their Subsets

Among the set of discovered SNP combinations discovered by DPM, some have better discriminative power than their corresponding subsets (like 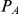 in Figure 1) while some have similar or lower discriminative power (like 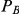 in Figure 1). A simple way to quantify the increase of discriminative power of a SNP combination over its subsets is to take a difference between the discriminative power of a SNP combination itself and the best discriminative power among all of its subsets. With the 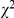 statistic as the measure for discriminative power, this difference (denoted as 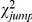) for a pattern 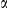 can be formally written as below. Note that, the 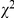 statistics of patterns of different sizes all have the degree of freedom of 1 based on the binary encoding of a SNP combination presented earlier in this section. Also note that, among the thresholds we used for 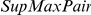 in the paper, the lowest is 0.15. This implies that the minimum frequency of any discovered SNP combination is 15% of the number of cases or controls (refer to the definition of 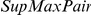 in the method section). Thus, the estimation of 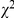 statistic for any SNP combination would be based on a frequency of at least 15% of the number of cases or controls, even for high-order combinations.
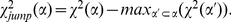
(1)


With the above definition, the 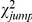 of the two patterns shown in Figure 1 are 14.4 and −6.1 and the four patterns in Figure S2 all have positive 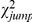 values (47.7, 14.4, 6.2 and 4.0 respectively). Indeed, 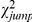 is not a new concept and similar measures based on other statistics for discriminative power (instead of 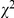 statistic) have been studied in data mining literature [56]. More generally, existing measures of epistasis and genetic interaction [39], [45] which capture the difference between the joint statistic between a SNP combination and the linear (or independent) addition of the its subsets, could be used for the same purpose as well. However, they are not suitable for high-order combinations analysis due to their increasing degrees of freedom and computational expense as combination size increases, which thus requires an increasing number of samples for accurate estimation. In contrast, 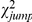 or similar measures based on other statistics have the advantage of a fixed degree of freedom (1) and thus are more practical for measuring the association between high-order combinations and a phenotype. Furthermore, the requirement of epistasis measures is more restrictive than measures like 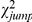 because the former only captures non-additive effect while the latter targets the general combined effect including both linear and non-linear combinations. Indeed, as will be shown in the result section, both linear and non-linear high-order combinations exist in real datasets, and both can be highly discriminative with respect to a disease phenotype and thus are of great interest.

Intuitively, it would be ideal if an algorithm like SMP can directly differentiate combinations with positive and negative values and then prune the ones with negative values as early as possible in the searching process. However, this is a non-trivial task because the 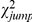 does not have the antimonotonicity property (crucial for the efficient enumeration of high-order combinations using the Apriori strategy [47]) and thus some combinations with large positive 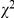 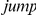 would be missed if they have subsets with negative 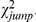 Therefore, in this study, we use SMP to first discover a set of discriminative combinations and then apply a 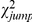 based filtering as a separate step.

### Many High-order Patterns are Trivial Extensions of their Smaller Subsets

**Figure 2:**
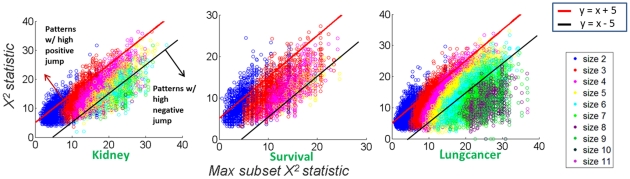
Comparing the 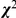 statistic of each pattern with the maximal 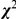 statistic among all of its subsets. The three subfigures correspond to the three datasets. Each subfigure shows the 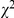 statistic of each pattern and the maximal 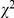 statistic among all of its subsets for all the discovered patterns. The color of a circle indicates the size of the pattern. The red line and the black line in each subfigure show 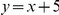 and 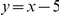 respectively.

We ran DPM on the three real datasets (with 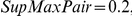 the lowest threshold that DPM can finish within 0.5 hour) and produced a set of SNP combinations from each dataset. With the three sets of discovered patterns, we first study a key mathematical property of high-order patterns, that is, if these combinations provide additional insights beyond their subsets. Specifically, for each combination, we calculate its 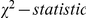 and 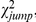 and summarize the results in Figure 2, with the three subfigures corresponding to the three datasets. Each subfigure shows the 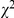 statistic of each pattern and the maximal 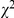 statistic among all of its subsets, for all the discovered patterns. The 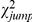 thresholds of +5 and –5 are indicated by a red line and a black line respectively, in each subfigure. Clearly, many large size patterns have negative 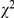 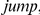 which indicates that many high-order patterns are trivial extensions of their smaller subsets (such as pattern 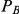 in Figure 1). They are not interesting or at least not informative for either enhancing the predictive power of a pattern or exploring functional interactions among the patterns in a SNP. Note that, +5 and –5 are used as two threshold of 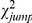 in Figure 2 just for visualization purpose, while different thresholds are studied in the separate experiments.

### Some High-order Patterns are Highly Discriminative Beyond Univariate and Low-order SNP-combinations

We also note that there are indeed several high-order combinations that provide higher discriminative power than any of their corresponding subsets. Specifically, in the datasets, Kidney and Lungcancer, there are tens of size-4 and size-5 patterns above the line of 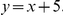 These patterns may indicate high-order functional gene interactions whose joint genetic variations result in a stronger association with the disease phenotypes than singletons and lower-order combinations. Again, +5 and –5 are used as two threshold of 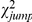 in Figure 2 just for visualization purpose, while different thresholds are studied in separate experiments. The observation that only a small fraction of high-order patterns have large 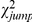 values motivates the design of targeted search algorithms that specifically look for patterns with large 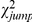 in addition to high 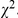 However, this is a non-trivial task as discussed in the method section.

Many patterns with high 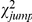 (e.g. above the line of 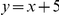) in the three datasets have 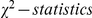 greater than 20, which corresponds to a low p-value of 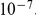 However, because a huge number of hypotheses were tested in the SMP search, we need to correct for multiple hypothesis testing. We use a permutation-test based approach (see methods section) to estimate unbiased and reliable false discovery rates (FDRs) for the patterns discovered and shown in Figure 2 (methods section).

**Figure 3:**
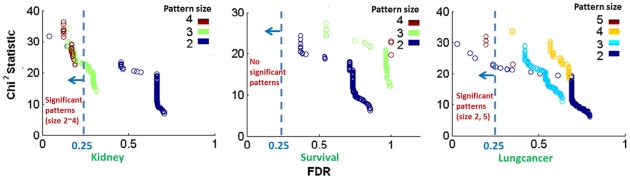
The 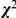 statistics and FDRs for the patterns with 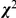 *jump* above 5. The layout follows that of Figure 2. In each subfigure, each circle is a pattern with the color indicating pattern size. Y-axis is the 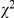 statistic of a pattern of size-*k*, and X-axis shows its permutation test-based FDR, which is size-specific as described in the method section.

Figure 3 shows the 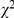 statistics and FDRs for the patterns with 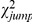 above 5 (different parameters for 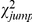 are studied in separate experiments), with a layout similar to Figure 2. The circles with similar color are clustered together, which results from the size-specific permutation tests which estimate the FDR of a size-*k* pattern from the null distribution built with only the random patterns of size *k* (see method). We observe that there are several significant patterns with FDR (w.r.t. 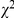) below 0.25 discovered from the datasets Kidney (up to size-4) and Lungcancer (up to size-5). Note that in Figure 3, we only consider the patterns with high 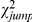 (above the line of 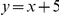). We will present a separate experiment that illustrates the benefit to statistical power of using 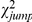 based filtering where we try different thresholds of 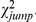


To better understand the effect of sample size on the FDRs of the patterns discovered from the real datasets. We designed an experiment with the same synthetic datasets used in the scalability test (Figure S3). Specifically, we examine the effect of sample size on the FDRs of the four embedded synthetic SNP combinations of sizes 3, 4, 5 and 6, respectively. Table S2 summarizes the FDRs of each pattern in each synthetic datasets with different sample sizes. The key observation is that, although the FDRs of embedded patterns are expected to be more significant when the sample size increases, all the four synthetic patterns have perfect FDR (<0.002, i.e. no better patterns were found in any of the 500 permutations), when the sample size is above 200. This indicates that the sample sizes in the two real datasets (Lungcancer and Kidney) are expected to be good enough for high-order SNP combination search. However, when the sample size is below 200, two of the four embedded real patterns (the size-4 one and size-5 one) can not be discovered with significant FDRs. This is also consistent with our observation on the other real datasets (Survival), on which no significant SNP combinations were discovered. Therefore, this new experiments helped the understanding of the effect of sample sizes on FDR and also support the statistical reliability of the patterns discovered from the two real datasets.

### Two Procedures that Generally Enhance Statistical Power of High-order SNP Combination Discovery

Here, we present the results studying two procedures for reducing the number of hypothesis tests in DPM, and their effect on enhancing the statistical power of high-order SNP combination discovery. The two procedures are: (P1) enforcing a proper threshold of 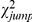 and (P2) using gene-set (e.g. pathway) constraints. They both have been used in existing literature for improving the computational efficiency of a combinatorial search framework [38], [45]. However, their effect on improving statistical power has not been systematically studied. The scalability of DPM for discovering high-order SNP combinations provides an opportunity to explore this. The statistical power is indirectly measured by the number of combinations and unique SNPs discovered with respect to a specific false discovery rate of 0.25. (Although somewhat arbitrary, a cutoff is needed. We choose a relatively high FDR threshold as in [24] because, for high-order SNP combination discovery which is still at its early stage, the research focus is more about hypothesis generation instead of hypothesis verification).

#### Use of 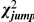 based filtering generally improves statistical power

**Figure 4:**
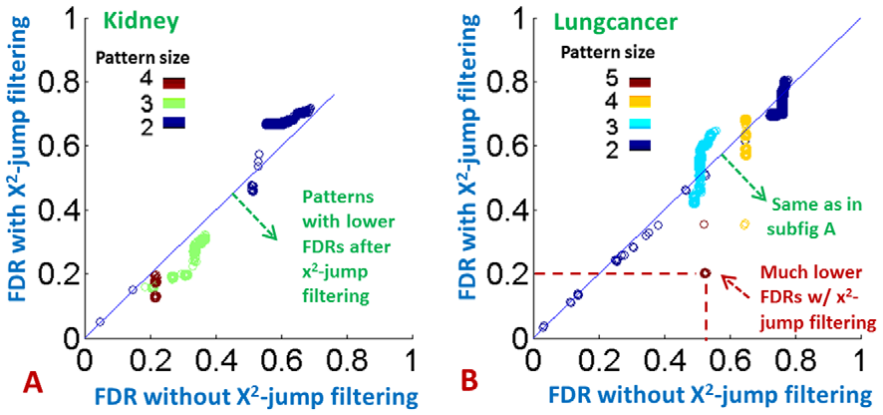
Comparison between the FDRs without 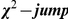 based filtering and the FDRs with 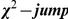 filtering for the Lungcancer and Kidney datasets respectively. In both subfigures, each circle represent a SNP combination. There are several circles sitting below the line 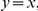 indicating that they have lower (more significant) FDR when a 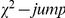 filtering is applied compared to the case where no 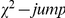 is used.

In Figure 3, the FDRs are estimated only with those patterns having sufficiently high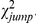 Here, we study whether using a 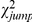 based pattern filtering improves the statistical power of the framework. Figure 4 (each circle represent a SNP combination) compares the FDRs without 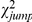 based filtering (x-axis) and the FDRs with 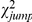 filtering (y-axis) for the Lungcancer (left subfigure) and Kidney (right subfigure) datasets. We tried three different thresholds for 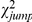 (0, 3 and 5) and found that the results are similar, which suggest the essential effect of the filtering is to eliminate those patterns with low negative 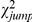 values. The figures shown here are based a threshold of 5 for 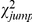. We use these two datasets for this comparison because there are more high-order combinations with high 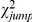 discovered from them (up to size-4 and size-5) and because none of the pattern discovered from the other dataset (Survival) have FDR (w.r.t. 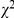) below 0.25. In both subfigures, there are several circles sitting below the line 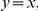 indicating that these patterns have lower (more significant) FDR (w.r.t. 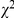) when a 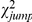 filtering was applied compared to the case where 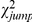 was not used. Specifically, there are seven combinations in the right subfigure (the red ones indicated by the arrow) which have an insignificant FDR (0.5) when no 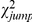-based filtering was applied, but low FDRs (around 0.2) when a 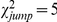 filtering was used.

This comparison demonstrates that 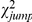 can enhance the statistical power of discriminative SNP-combination discovery and potentially discover SNP combinations that would have been missed. This can be explained as follows: for a real pattern *P* of size-k and a high 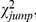 the use of 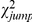 filters out random patterns in the permutation tests that have high discriminative power but are trivial extensions of its subsets, which would otherwise penalize the statistical significance of 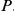 Essentially, the use of 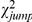 based filtering provides a better estimation of the statistical significance of a pattern with high 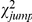 by estimating a more reasonable null distribution.

As discussed earlier, 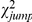 is just one of many possible measures that quantitatively describes the increment of discriminative power of a pattern with respect to its subsets. Specifically, the 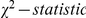 can be replaced by other measures of discriminative power, or the difference can also be replaced by measures for statistical epistasis [39], [45]. The observations from the above comparison, where 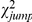 is used as a representative, supports the use of these measures to improve the statistical power of discriminative SNP combination discovery.

#### Applying gene-set constraints generally improves statistical power

As discussed in the introduction, gene-set constraints can reduce the number of hypothesis tests and thus have the potential to enhance the statistical power of high-order SNP combination search. However, the reduction of search space based on prior knowledge also risks missing novel combinations that are not supported by known gene sets. This calls for a quantitative estimation of the tradeoff. Leveraging the efficiency and scalability of the proposed framework, we design the following experiments to explore how gene-set constraints improve the statistical power of high-order SNP combination search (see methods section about how to incorporate gene-set constraints in DPM), where the power is measured indirectly by the number of combinations and unique SNPs discovered with respect to a false discovery rate based on permutation tests as described in the method section. We use the 1892 gene sets from the Molecular Signature Database (MSigDB, 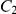) [24] as the source of biological constraints.

Table 1 summarizes the comparison we designed on the three real datasets. We compared the without-constraint setup (*A*) with two variations of with-constraint setups, one with a 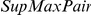 threshold that is the same with setup *A* (0.2, the lowest threshold that DPM can finish within 0.5 hour without gene-set constraints) and the other with a threshold (0.1, the lowest threshold that DPM can finish within 0.5 hour with gene-set constraints) that is lower to demonstrate that the gain of computational efficiency with gene-set constraints allows the search for combinations with lower frequency. The latter two setups are denoted as *B* and *C* respectively. To study how the size of gene sets affects the statistical power of the proposed framework, we use a parameter (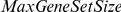) to select the gene sets to use in each experiment. Specifically, for each dataset, we conducted the experiments in *B* and *C* with 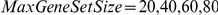 and 100 respectively. Note that, we only vary 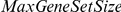 below 100 because we observed that when gene sets have more than 100 genes, there are few if any statistically significant (with FDR (w.r.t. 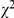) below 0.25) SNP combinations (with respect to permutation-based FDRs). Several key observations can be made from Table 1.

**Table 1:** Parameters used and number of significant patterns discovered for each of the three datasets, for evaluating the effects of gene-set constraints on enhancing statistical power after correcting multiple hypothesis tests.

Data Name	Exp NO.	Gene Set Constrains	Patt Size	MaxGeneSetSize				
				20	40	60	80	100
Kidney	A	N	2	2(3)				
			3	64(61)				
			4	34(50)				
	B	Y	2	2(3,3)	0	0	0	0
	C	Y	2	0	0	0	6 (10,5)	0
Survival	A	N		0				
	B	Y	2	2(3,3)	2(3,3)	2(3,3)	5(8,8)	3(5,5)
	C	Y	2	5(7,7)	11(14,14)	7(10,10)	7(10,10)	11(17,17)
Lungcancer	A	N	2	14(12)				
			5	7(12)				
	B	Y	2	12(10,7)	0	0	0	0
			3	0	0	0	6(10,10)	8(16,13)
	C	Y	2	0	4(6,6)	4(6,6)	5(8,8)	0

Parameters used and number of significant patterns discovered with respect to the FDR cutoff 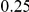 for each of the three approaches (A: without constraint, 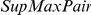 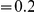; B: with constraints, 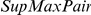 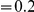 and C: with constraints, 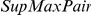 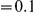) on each of the four real datasets. The number outside the brackets are the number of significant patterns discovered, and the first number inside a bracket shows the number of unique SNPs covered by the patterns (note that there are overlaps between patterns); the second number inside the s bracket (for approaches B and C only) indicates the number of SNPs that are discovered by approaches B or C but not by approach A in the corresponding dataset, thus indicating the benefit of using gene-set constraints.

A key observation is that, gene-set constraints are generally effective for improving the statistical power of high-order SNP combination discovery. For Survival, none of the discovered combinations have FDR (w.r.t. 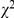) below 0.25 in the without-constraint setup. In contrast, with the gene-set constraints, there are tens of significant (with FDR (w.r.t. 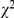) below 0.25) SNP combinations discovered (all of size-2). On the other two datasets, although the without-constraint setup discovers more significant combinations than the with-constraint setups, additional SNPs can be discovered in the with-constraint setups, as indicated by the second numbers in the brackets.

However, gene-set constraints sometimes can miss interesting SNP combinations. For the dataset Kidney, without-constraint setup discovers 98 statistically significant SNP combinations (with FDR (w.r.t. 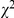) below 0.25) of sizes 3 and 4 (permutation-test based FDR less than 0.25 after correcting for multiple hypothesis tests), while the two with-constraint setups only discover 2 and 6 combinations with FDR (w.r.t. 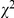) below 0.25 (all of size 2), respectively. The possible explanation is that the gene sets in MSigDB 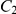 may not describe the functional pathways related to the phenotype in the Kidney dataset (rejection vs. no-rejection for the patients with kidney transplant). This observation indicates that the effectiveness of gene-set constraints depends on the gene sets used and varies from phenotype to phenotype.

A final observation is that, setup *C* (with-constraint using lower 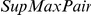) allows the search of lower-frequency SNP combinations. Specifically, on Kidney and Survival, more significant SNP combinations with FDR (w.r.t. 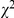) below 0.25 are discovered when the lower 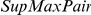 (0.2) is used. This demonstrates the existence of low-frequency yet statistically significant SNP combinations and thus the benefits of searching low-support SNP-combinations, which is enabled by using gene-set constraints.

### Exploring Functional Interactions in High-order Combinations

Existing work that studies functional interactions in SNP combinations mostly focuses pairs of loci [18], [57]–[60]. The few studies that explored functional interactions in high-order combinations are mostly based on SNP datasets that cover a small number of genes [17]. In addition, these studies only focus on one or a few top ranked combinations discovered from a single dataset and thus only reveal disease-specific functional interactions [17], [41]. In this study, before interpreting the top high-order SNP combinations, we first explore functional interactions in SNP combinations from a more general perspective. The aim is to exploit some common insights on functional interactions in discriminative SNP combinations consistent across multiple datasets which may provide some guidance for future studies.

#### Positive connection between 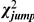 and within-pattern functional coherence

Specifically, we study how the increase of discriminative power of a SNP combination over its subsets is related to the functional coherence of the genes covered by the combination. For this purpose, we divide all the discriminative patterns discovered by SMP into three groups, i.e. those having 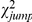 values in 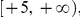 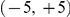 and 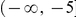 (denoted as 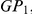 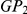 and 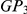 respectively) and study the relative functional coherence of the patterns in the three groups. To measure the functional coherence of a SNP combination, we first obtain the set of genes covered by the combination by assigning a SNP to its closest gene, and then determine the functional similarity between each unique pair of genes covered by the combination using a human functional network integrated from a comprehensive set of resources [61]. Essentially, such an estimation decomposes the functional coherence of a set of genes covered by a SNP combination into the functional similarities of the set of unique gene pairs. We prefer this approach to a GO enrichment analysis [62] because: 1) the former can provide more detailed functional insights on gene-gene interactions within high order combinations, and 2) the latter is usually applicable to gene sets that are of sizes larger than the high-order SNP combinations discovered in this study (size-3, 4 or 5, Figure 3). With the decomposition-based approach for each SNP combination, we can get three distributions of gene-gene functional similarities for the three groups of SNP combinations 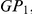 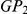 and 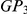 respectively, where each distribution contains the functional similarities of the union (unique) of the within-pattern gene pairs from all the patterns in one of the three groups. In addition to the three distributions, we also generate a null distribution (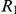) by repeating the following procedure 100 times: we randomly sample gene pairs from the set of genes covered in the corresponding dataset as many as the number of gene pairs in 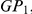 while fixing the number of times each unique gene occurs with respect to 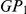. Because we binarize the human functional network [61] at 0.5 (The corresponding network has a density of 5%) to make the size of the network efficient to manage). It is worth noting that the following results are consistent across different cutoff values for the functional network (0.5, 0.6, 0.7 and 0.8).

**Figure 5:**
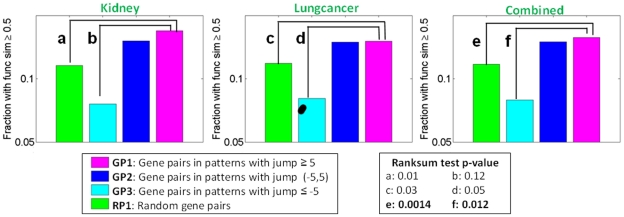
Functional similarity of within-combination gene pairs in three groups of discriminative SNP combinations and the null distributions (best view in color). This is to reveal the connection between 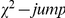 and within-combination functional coherence. The six comparisons, 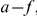 and the associated ranksum test p-values are also shown.

Figure 5 summarizes the comparison among the four distributions in term of the fraction of functional similarities above 0.5 and the p-values of the ranksum tests for (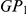 vs. 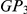) and (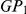 vs. 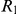). The comparisons are done on the Kidney and Lungcancer dataset but not on Survival because there are significant SNP combinations (with FDR (w.r.t. 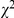) below 0.25) discovered on the former two but not the latter as shown in Figure 3. A key observation is that 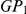 has higher within-pattern functional similarity than both 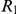 and 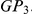 This is reflected by the consistently higher fraction of within-pattern gene pairs with functional similarity scores above 0.5 in 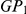 than in 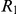 and 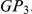 The relative order among 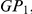 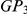 and 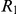 is significant (ranksum test p values as shown in the figure) and consistent on the datasets Kidney and Lungcancer as well as the combined. (Datasets Kidney and Lungcancer cover the same set of genes and thus have the same null distribution of gene-pair functional similarity. Therefore, we combine the each of the four sets of gene pairs (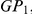 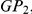 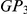 and 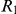) from the two datasets to increase the sample size and allow a more reliable estimation of p value.) This observation provides a novel positive connection between the increase of discriminative power of a SNP combination over its subsets and the functional coherence among the genes covered by the combination. Essentially, this set of observations suggest that 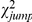 not only improves the statistical power of the discriminative SNP-combinations search framework (as shown in earlier), it is also indicative on the biological relevance of the genes covered by discriminative SNP combinations. The fact that 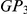 has the lowest fraction of functional scores above 0.5 further supports that a 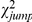-based filtering is helpful and important for further exploration of functional insights from discriminative SNP combinations. The results are consistent across different cutoff values for the functional network (0.5, 0.6, 0.7 and 0.8).

It is worth noting that, 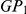 and 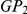 have about the same fraction of functional scores above 0.5 on both Kidney and Lungcancer and the ranksum tests between them are insignificant (ranksum test) on both datasets. This suggests that the genes covered by a SNP combination with 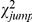 around zero also tend to be functionally related. This may be explained by existing study on positive yeast genetic interactions [63] where multiple genetic perturbations targeted on a single pathway are often found to have similar effect as the genetic perturbation of just one gene in the pathway. In contrast, the SNP combinations with 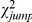 highly above zero (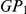) may correspond to the genes that are involved with multiple pathways that have compensation with each other, or correspond to the genes on a single pathway but with dosage effect [63]. To our knowledge, this set of analysis is the first exploring the connection between discriminative power of SNP combinations and functional interactions from a general perspective across multiple datasets.

#### Specific interpretation of two patterns discovered from Datasets Lungcancer and Kidney

**Figure 6:**
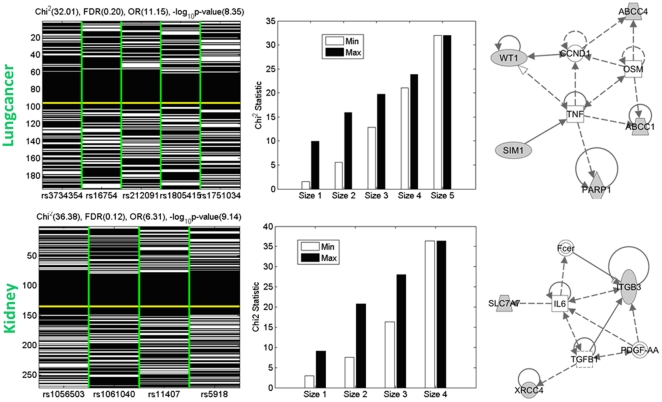
Visualization of two SNP-genotype combinations discovered from the Lungcancer and Kidney datasets respectively. The interpretation is similar to the subfigures in Figure 5. The rsnumbers of the five SNPs in the Lungcancer pattern and the four SNPs in the Kidney pattern (all with *MM* genotype) are shown. The SNPs in the two patterns are mapped to the following two sets of genes, (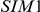, 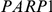, 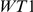, 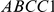, 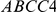) and (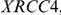 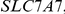 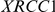 and 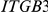). The 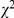 statistics of the pattern and its subsets are shown in the right subfigures. Their permutation test-based FDRs and odds ratios are also shown. The top enriched molecular interaction network (by Ingenuity Pathway Analysis) is also shown for each pattern, where the shaded nodes are those genes mapped from the SNPs in each pattern.

Beyond the above general biological insights, we also find that several high-order patterns with high 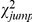 that are biologically interesting with respective to the complex diseases, e.g. size-5 patterns in the Lungcancer dataset and size-4 patterns in the kidney dataset. Figure 6 illustrates two examples with descriptions similar to Figure 1: a size-5 pattern discovered from Lungcancer with an odds ratio of 11.15, an p-value of 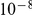 and a false discovery rate of 0.20, and a size-4 pattern discovered from Kidney with an odds ratio of 6.31, an p-value of 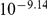 and a false discovery rate of 0.21. It is interesting that the two patterns are both more discriminative than their subsets. Furthermore, we also found that the *synergy*, a measure of statistical epistasis capturing non-additive interactions [45], of the Lungcancer pattern is positive, indicating a probable interaction beyond additive effect.

The five SNPs in the Lungcancer pattern are mapped to the five genes that are closest (chromosome location) to them respectively, 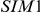 [MIM: 63128], 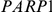 [MIM: 173870], 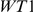 [MIM: 607102], 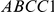 [MIM: 158343] and 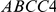 [MIM: 605250]. Four out of the five genes (the latter four) are previously known to be associated with cancer, with the latter three being associated with lung cancer specifically [64]–[66]. 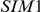 has been shown to interact with 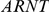 [MIM: 126110], which binds to Aryl Hydrocarbon Receptor (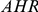 [MIM: 600253]), and the 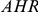 pathway has been recently shown to be activated upon binding of various exogenous chemicals from cigarette smoke and might link to lung cancer risk [67]. 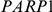 is a poly(ADP-ribose) polymerases-1, involved with DNA repair and has been associated with both better survival in non-small cell lung cancer, as well as with increased risk of lung cancer [68]. 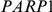 is becoming an important target for cancer therapy, as inhibitors of PARP have low toxicity [69]. There was also a group that showed that transcriptional activation of 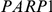 leads to in-silica malignant transformation of human bronchial epithelial cells [70]. 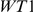 (Wilms tumor 1) has been shown to be a critical regulator of senescence and proliferation downstream of oncogenic 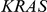 signaling [71], and 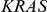 [MIM: 190070] is one of the most frequently mutated human oncogenes. 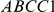 and 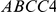 are ATP-binding cassette genes, sub-family C, involved with multi-drug resistance [72] so their association with lung cancer here might have something to do with therapy. Discovering these five SNPs together as a highly predictive combination with an odds ratio of 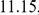 and a large 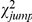 of 7.9 may provide novel insights on their combined effects (beyond their separate effects) on their association with lung cancer. The top molecular interaction network (using Ingenuity Pathway Analysis (http://www.ingenuity.com, accessed 2012 Feb 20)) for this Lungcancer pattern is also shown. This molecular subnetwork is associated with cell death and cell cycle function with the top enriched disease being cancer, and therefore supports the functional interaction among the set of genes and their joint association with the risk of lung cancer. The other pattern in Figure 6 (size-4) is discovered on the Kidney dataset (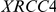 [MIM: 194363], 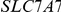 [MIM: 603593], 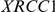 [MIM: 194360] and 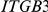 [MIM: 173470]). The four gene corresponding to the four SNPs are also enriched with a molecular interaction network with annotations closely related to transplant after kidney transplant: organismal injury and abnormalities, cellular movement, cellular-mediated immune response and cellular development.

It is worth noting that such statistically significant and biologically relevant discriminative SNP combinations are mostly high-order combinations of common variants (those SNPs with high allele frequencies). While the current focus in the exploration of the genetics of rare diseases is mostly on individually rare variants, these high-order SNP combinations indicate that common variants could also be the cause of rare diseases because combinations of common variants can be a rare composite variant.

## Discussion

We presented a computational framework for searching high-order SNP combinations with strong disease association from case-control datasets with thousands of SNPs. The framework is substantially more efficient and scalable than existing techniques that usually handle tens of or hundreds of SNPs and mostly up to size-3 combinations. We further showed that, while most high-order combinations are trivial extensions of their subsets, there are indeed high-order combinations in real datasets and they have stronger associations with some disease phenotypes beyond single SNPs and low-order SNP combinations. We also evaluated the effect of two strategies for enhancing the statistical power of high-order SNP combination search: filtering out SNP combinations with lower or similar discriminative power than their subsets and constraining the search space with known biological gene sets. Further leveraging the improved statistical power of this framework, we explored the functional interactions within the SNP combinations discovered from three real case-control datasets and revealed a positive connection between the increase of discriminative power of a SNP combination over its subsets and the functional coherence among the genes covered by the combination. Last but not least, we investigated two representative high-order SNP combinations (one of size-5 and the other of size-4) discovered from a lung cancer case-control dataset and a kidney transplant-rejection case-control dataset respectively, and showed that the genes covered by the two patterns are enriched with molecular interaction networks that are highly relevant to the risk of lung cancer and risk of rejection after kidney transplant, respectively. These results demonstrate the ability of our approach to find statistically significant and biologically relevant high-order, patterns, but we likely find only a subset of all possible SNP patterns of interest. In particular, some interesting patterns could be eliminated during the discriminative pattern mining step or in the 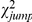 filtering step. Other existing approaches may discover some of these missed patterns, but likely miss many of the high-order patterns we find. Thus, what we provide is a well-founded and efficient (even though not complete) approach to pattern discovery in SNP datasets.

Given that there has been a lack of tools for higher-order combination analysis due to computational and statistical challenges, the proposed framework is expected to help discover novel genotype-phenotype associations missed by existing approaches that mostly take the route of univariate analysis, pathway/network enrichment analyses that are based on univariate statistics, or epistasis analysis of low-order SNP combinations. In addition to the proposed framework itself, some general observations made in this study could also help the development of other computational techniques that search for high-order SNP combination and exploit functional insights, namely: 1) two strategies for enhancing statistical power to cope with multiple hypothesis testing in the combinatorial search could be leveraged by other approaches, 2) the observed positive connection between the increase of discriminative power of a combination beyond its subsets and the within-pattern functional coherence, both of which may guide more comprehensive exploration of functional insights of high-order interaction, and 3) the observation that many significant associations are rare combinations of common variations, which suggests an alternative direction to explore the genetics of rare diseases for which current focus is on individually rare variations.

The three real datasets used in this paper represent a type of studies that have a different perspective from the typical disease-control designs used in most genome-wide association studies (GWAS). Specifically, the case-control designs used in the three studies are the short vs. long survival of multiple myeloma patients (all received the same treatment), acute rejection after kidney transplant (all received the same treatment) and patients with lung cancer and normal subjects (all heavy smokers). Studies with such or similar designs enforce strict additional criteria in sample selection and thus normally have much fewer samples compared to most GWAS studies. Given the limited sample sizes, the three studies adopted a SNP chip that targets a set of SNPs selected on the basis of biological candidacy in order to have better statistical power. Therefore, we expect the proposed framework to help other studies that also use targeted SNP chips to search for high-order SNP combinations that provide insights beyond univariate or lower-order analysis.

The proposed framework is able to efficiently search high-order combinations for focused studies with thousands of SNPs, but not directly suitable for focus studies with even more SNPs (e.g. tens of thousands) or genome-wide data. However, note that, this limitation is not specific to the proposed approach but to high-order interaction discovery in general, because there it is computationally infeasible to search for high-order interactions directly from genome-wide SNP datasets. After all, the state of the art methods for discovering high-order interactions could only handle less than a thousand SNPs as reviewed in the paper. Nevertheless, a practical solution to handle genome-wide datasets is to apply the current framework on a subset of SNPs selected by some prioritization strategy [7], e.g. adopt tag SNP selection [50] techniques to first obtain a set of less redundant SNPs, or only search for high-order interactions involving those that have sufficient marginal effects as done in [38], [39], or only search for high-order interactions among the SNPs within a certain category based on prior biological knowledge, e.g. a pathway or a genomic region, etc.

There are several possibilities for future work. First, we used a binary encoding for SNP-genotype combinations which differentiates the present of all the SNP genotypes in a pattern in a subject from the mismatch of any one genotype, but not further distinguish different numbers of mismatches. A more generalized encoding [73] that reflect the numbers of mismatches can be incorporated into the DPM framework and further explored. Second, the current study only assigns a SNP to the closest gene when exploring the functional similarity within a SNP combination, and thus may ignore the effect of a SNP on affected genes located far from the SNP (e.g. long distance cis-regulation or trans-regulation). In future work, one could integrate SNP data with gene expression data (when available) to map eQTLs before studying functional interactions within a SNP combination [74]. Third, because the current framework cannot automatically handle datasets with a large imbalance of race or gender between cases and controls, we only analyzed datasets with balanced or slightly imbalanced populations by requiring a large minimum differentiation threshold and only considered autosomal SNPs in order to avoid trivial discoveries. To make the current framework more widely applicable, we could select a subset of cases and controls to enforce a balance of population structure based on genome-wide autosomal clustering [26] or we could explore some generalization approaches that have been used to allow MDR to automatically handle confounding factors and continuous traits [18]. Last but not least, although this study focused on the discovery of high-order combinations from SNP datasets, a similar framework could also be applied for discovering combinations of other formats of genetic variations such as copy number variations or epigenetic variations such as DNA methylation, or even more generally across different types of (epi)genetic variations.

## Materials and Methods

### Three SNP Datasets and Pre-processing Considerations

We carefully checked the race and gender information in the three datasets to make sure the high-order combinations are not due to spurious allelic association as suggested by [75], [76]. Specifically, the subjects in the first two datasets are all Caucasian descendants and the last dataset contains both Caucasian and African American samples with an 9% imbalance between the cases and controls. We require the minimum differentiation between cases and controls to be 15% in all the SNP-combination search and analysis, in order to avoid the discovery of trivial difference due to population substructure, and we only consider SNPs from autosomes to remove the effect of gender imbalance. As shown in the result section, the comprehensive functional analysis on the discovered SNP patterns also supports that the discovered SNP combinations are functionally related to the disease instead of confounding factors such as gender and race. SNPs with more than 5% missing values are also removed.

### Simulation of a Synthetic Case-control SNP Dataset

We first used Hap-Sample simulator (http://www.hapsample.org, accessed 2012 Feb 20) to simulate genotype data with the 3404 SNPs from a recent study on multiple myeloma [34] as input, out of which 2172 SNPs are included in Hap-Sample. The synthetic dataset contains 70 cases and 70 controls (randomly generated from the HapMap project [77]). Note that this genotype dataset by itself does not contain disease-associated loci. Therefore, as a proof of concept we further embedded four synthetic high-order SNP combinations of size 3,4,5 and 6 respectively, that are associated with the case-control grouping, as shown in Figure S2 (with similar description as those shown in Figure 1). To study the scalability of SMP with respect to sample size (summarized in Figure S3), we further generated another 6 synthetic datasets with sample size (cases and controls combined) from 280 to 5600 in seven steps (280, 420, 560, 1400, 2800 and 5600). In each of the additional six datasets, we first use Hap-Sample simulator to generate SNP genotypes for more samples. Then, we embedded the same four synthetic patterns as done in the first data but increasing the number of samples while maintaining the frequency of each SNP genotype in the cases and controls. All the synthetic datasets are available from the supplementary website.

### The Apriori Framework: Efficient Combinatorial Search with Anti-monotonic Objective Function

The Apriori framework is essentially a bottom-up exhaustive combinatorial search framework initially designed for association analysis on binary data. It first searches all the size-2 combinations and then moves up to size-3 and so on. Different from brute-force search, the Apriori framework leverages the antimonotonicity of the objective function for pruning the combinatorial search space. Specifically, an objective function *F* is anti-monotonic if the following equation holds:
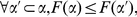
(2)where 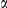 is any combination of SNPs with the binary encoding described in the result section. An anti-monotonic objective function can be used in the Apriori algorithm to efficiently traverse the combinatorial search space without the need of visiting all the nodes in the search space, because as soon as 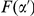 is found to be disqualified with respect to a threshold (*t*) (i.e. 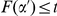), Apriori can prune all the supersets of 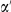 without missing any combination with an *F* value greater than *t*, given that the anti-monotonicity of *F* guarantees that 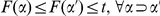. Further details on the optimized implementation of the Apriori framework can be found in [47].

### The Anti-monotonic Objective Function 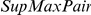


Given a case-control dataset, the 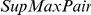 of a SNP combination 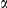 (with the binary encoding described in the result section) is defined in [48] as below (assuming the combination is more frequent in the cases; similarly for the other situation when the combination is more frequent in the controls):
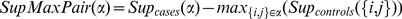
(3)where 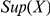 denotes the frequency (in percentage) of a SNP combination in a set of samples, cases or controls as shown in the subscript. So, 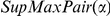 is defined as the difference between the frequency of a SNP combination in the cases and the maximal frequency of its size-2 subsets in the controls. An objective function defined in this way not only captures the frequency difference of a SNP combination between the cases and controls, but also has the antimonotonicity property, because the difference between an anti-monotonic function (frequency 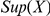) and a monotonic function *max* is anti-monotonic (refer to [48] for the formal proof).

Using 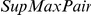 in the Apriori framework guarantees the discovery of all the SNP combinations that show at least some frequency differentiation between cases and controls on the size-2 level, as controlled by a threshold on 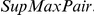 Therefore, if a size-5 combination does not show any differentiation until size-3 (or size-4, size-5) 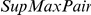 would miss it. As shown by a recent theoretical study [51], the possibility that a high-order (size-*k*) combination with strong differentiation shows zero differentiation in all of its subsets decreases dramatically when *k* increases (generally become impossible for *k* greater than 5). Therefore, in practice, we can use a threshold on 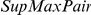 as low as possible (computationally more and more expensive) as long the computational time is still acceptable, in order to minimize the chance of missing interesting high-order interactions.

### Permutation Tests and Estimation of the False Discovery Rate (FDR)

Because of the large number of high-order SNP combinations tested in the search process, correction for multiple hypotheses testing is needed for a reasonable estimation of the statistical significance of the discovered SNP combinations. We follow the widely used empirical permutation-based approach (e.g. as used in [24]) to estimate false discovery rates (FDRs). Specifically, we first apply the proposed algorithm to the data with the original case-control grouping to get a set of discriminative patterns which are called the *real patterns*. Next, in each permutation test, we randomly shuffle the grouping of subjects into cases and controls while maintaining the original sample-size ratio between cases and controls, and then use SMP with exactly the same setup as for the original case-control grouping to discover a set of patterns. If 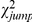 based filtering and gene set-based constraints are used for the original case-control grouping, the same procedures are also applied in each permutation in order to have an unbiased correction of multiple hypothesis testing. We repeat the permutation tests 100 times and get 100 lists of discriminative patterns which are called the *random patterns*. For each pattern (both real and random ones), we compute a 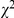 statistic. The false discovery rate (FDR) of a real pattern (with respect to a 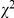 statistic of *c* and of size-*k*) is then calculated as follows: if there are *m* real patterns of size-*k* with 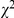 greater than *c* and there are *n* random patterns of size-*k* with 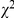 statistic greater than *c*, then the false discovery rate is 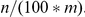 Note that, the run with real case-control label and each of the runs with randomized case-control label test the same number of hypotheses even though different number of combinations were pruned in the searching process.

Note that, in the above permutation based FDR computation, the estimation of FDR is specific to the pattern size. The use of size-specific FDR is motivated by the fact that it is harder and harder for a combination to provide additional discriminative power than all of its subsets as size increases. That is, given the same threshold of 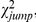 it is less likely to discover a larger combination than to discover a smaller one. This is supported by the observations made in Figure 2 as well as our recent work in [51] from a more theoretical perspective. In addition, this is in accord with the observations made by Ma et al. [33]. Therefore, we chose to estimate size-specific FDRs to better reflect the statistical significance of patterns of larger sizes. It is worth noting that estimating FDRs for combinations of different sizes separately might also increase the risk of discovering false positives. While one conservative approach is to do a second round of correction on multiple hypothesis testing over different combination sizes, we highlight the potential of discovering novel biological insights from a hypothesis generation perspective in this study. Indeed, the independent functional analyses presented in the result section with the discovered high-order combinations do support that the genes covered by the discovered combinations have significant functional relationship compared to the carefully controlled null distribution. Ma et al. [33] also proposed to include the subsets of a pattern for estimating its null distribution in addition to the random patterns discovered in the permutation tests. However in this paper, we estimate FDRs only with the random patterns discovered in the permutation tests because we directly enforce the requirement that a pattern has a sufficiently larger 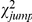 than its subsets.

### Applying Gene-set Constraints in DPM

Gene-set constraints have been used in some recent work [38] to improve computational efficiency and to make the biological interpretation of results easier. Essentially, a set of SNPs are considered for an association test only if the SNPs are located around the genes that are on a common pathway or interact with each other. Similar constraints can also be applied with molecular interaction networks, where the search is limited to a local subnetwork within a certain diameter. For example, human protein-protein interaction networks are used in in genome-wide SNP data analysis to reduce the search space of two-locus interactions [46]. It is worth noting that gene-set or molecular-subnetwork constraints also has antimonotonicity (if a set of SNPs do not belong to any gene set or molecular subnetwork, it is guaranteed that its supersets are not qualified either). Therefore, these constraints can be naturally incorporated together with 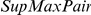 in the SMP framework to search for high-order patterns [48].

## Supporting Information

Figure S1 Transforming a toy SNP dataset in categorical representation to the corresponding binary representation.
(TIF)

Figure S2 Four synthetic discriminative patterns of size-36 that we embed in the synthetic dataset as described in the method section, with similar description as Figure 1.
(TIF)

Figure S3 The scalability of SMP with respect to sample size (cases and controls combined). The computational time of SMP increases linearly with the sample size (Note that the x-axis is not linearly spaced).
(TIF)

Table S1 Summary of the three real datasets. The second column lists the number of SNPs for each dataset after filtering out the SNPs with more than 5% missing values.
(DOC)

Table S2 The effect of sample sizes on the FDRs of the four synthetic SNP combinations (shown in Figure S2) embedded in the synthetic dataset.
(DOC)
