# Supplementary figures and images for: High-Order SNP Combinations Associated with Complex Diseases: Efficient Discovery, Statistical Power and Functional Interactions
Source: PLoS One. 2012 Apr 19;7(4):e33531. doi: 10.1371/journal.pone.0033531 (PMC3334940; doi:10.1371/journal.pone.0033531)

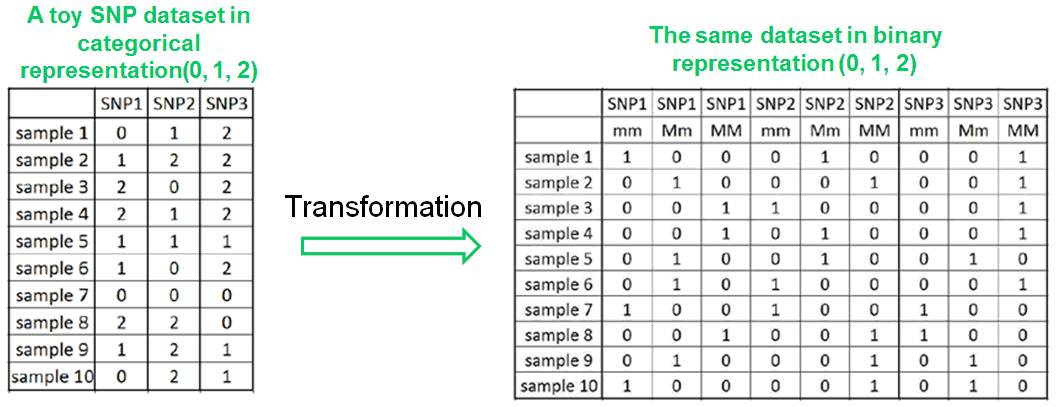

Supplement: Figure S1 — Transforming a toy SNP dataset in categorical representation to the corresponding binary representation. (TIF) [file pone.0033531.s001.tif]

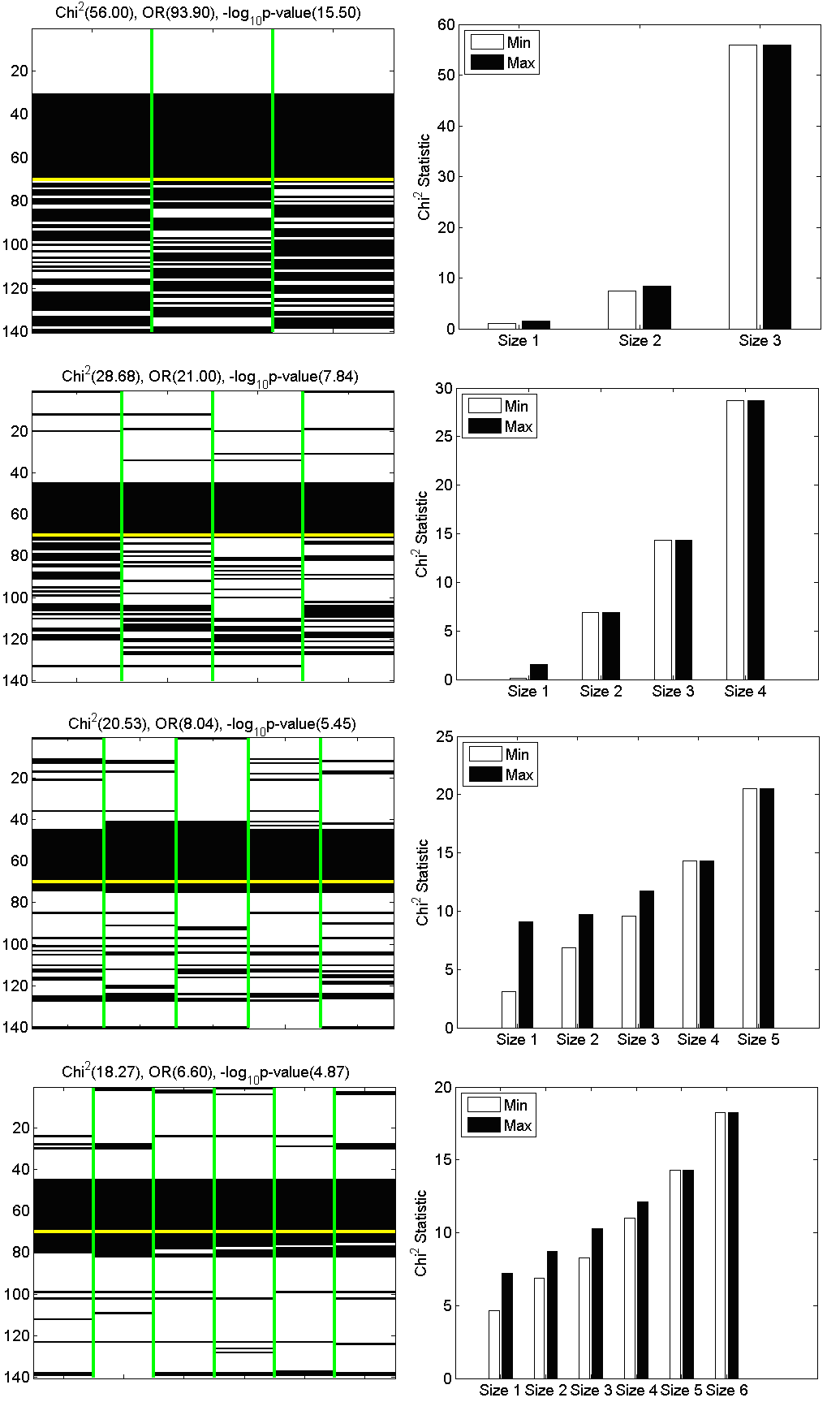

Supplement: Figure S2 — Four synthetic discriminative patterns of size-36 that we embed in the synthetic dataset as described in the method section, with similar description as Figure 1. (TIF) [file pone.0033531.s002.tif]

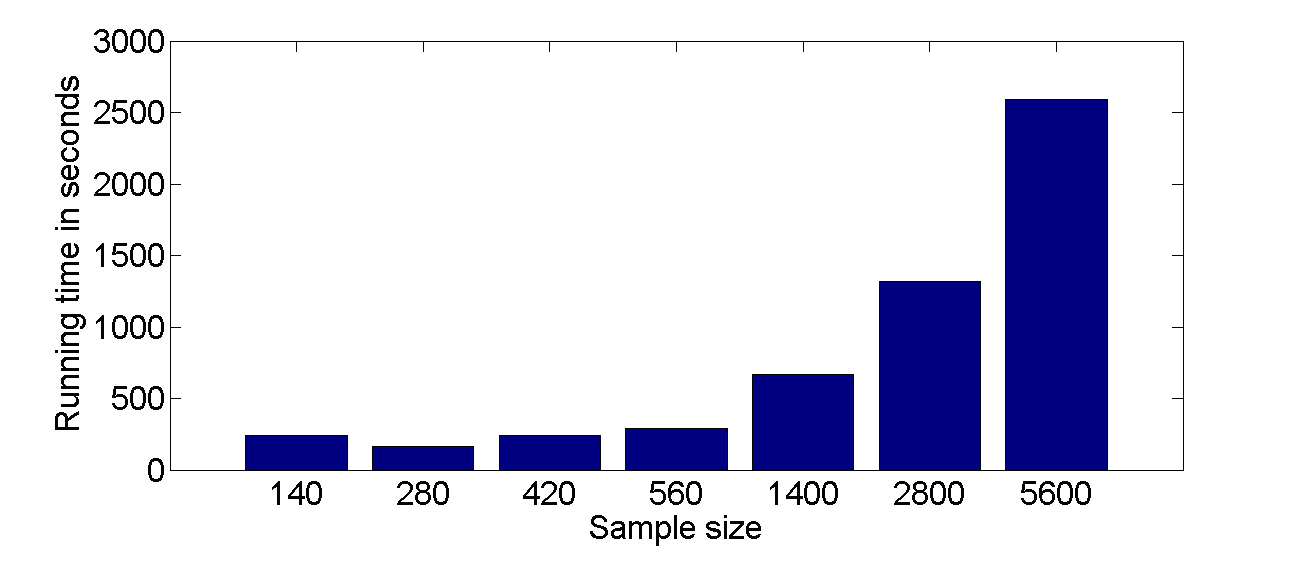

Supplement: Figure S3 — The scalability of SMP with respect to sample size (cases and controls combined). The computational time of SMP increases linearly with the sample size (Note that the x-axis is not linearly spaced). (TIF) [file pone.0033531.s003.tif]
