# Supplementary material for: High-Order SNP Combinations Associated with Complex Diseases: Efficient Discovery, Statistical Power and Functional Interactions
Source: PLoS One. 2012 Apr 19;7(4):e33531. doi: 10.1371/journal.pone.0033531 (PMC3334940; doi:10.1371/journal.pone.0033531)
Supplement: Table S1 — Summary of the three real datasets. The second column lists the number of SNPs for each dataset after filtering out the SNPs with more than 5% missing values. (DOC) [file pone.0033531.s004.doc]

**Table S1**. Summary of the three real datasets. The second column lists the number of SNPs for each dataset after filtering out the SNPs with more than 5% missing values.

| **Dataset** | **Number of SNPs** | **Number of Samples** | **Number of Cases** | **Number of Controls** |
| --- | --- | --- | --- | --- |
| ***Survival*** | 2755 | 143 | 70 | 73 |
| ***Lungcancer*** | 3428 | 195 | 96 | 99 |
| ***Kidney*** | 3394 | 271 | 135 | 136 |
