# Supplementary material for: High-Order SNP Combinations Associated with Complex Diseases: Efficient Discovery, Statistical Power and Functional Interactions
Source: PLoS One. 2012 Apr 19;7(4):e33531. doi: 10.1371/journal.pone.0033531 (PMC3334940; doi:10.1371/journal.pone.0033531)
Supplement: Table S2 — The effect of sample sizes on the FDRs of the four synthetic SNP combinations (shown in Figure S2) embedded in the synthetic dataset. (DOC) [file pone.0033531.s005.doc]

**Table S2**. The effect of sample sizes on the FDRs of the four synthetic SNP combinations (shown in Figure S2) embedded in the synthetic dataset. “< 0.002” indicates no better patterns were found in any of the 500 permutations.

|  | **FDRs of the four synthetic patterns** | | | |
| --- | --- | --- | --- | --- |
| Sample size (cases and controls combined) | Size-3 | Size-4 | Size-5 | Size-6 |
| 140 | <0.002 | 0.75 | 0.35 | 0.04 |
| 280 | <0.002 | <0.002 | <0.002 | <0.002 |
| 420 | <0.002 | <0.002 | <0.002 | <0.002 |
| 560 | <0.002 | <0.002 | <0.002 | <0.002 |
| 1400 | <0.002 | <0.002 | <0.002 | <0.002 |
| 2800 | <0.002 | <0.002 | <0.002 | <0.002 |
| 5600 | <0.002 | <0.002 | <0.002 | <0.002 |
